# Emotion or Evaluation: Cultural Differences in the Parental Socialization of Moral Judgement

**DOI:** 10.3389/fnhum.2022.867308

**Published:** 2022-06-10

**Authors:** Sawa Senzaki, Jason M. Cowell, Yuki Shimizu, Destany Calma-Birling

**Affiliations:** ^1^Department of Psychology, University of Wisconsin–Green Bay, Green Bay, WI, United States; ^2^Faculty of Letters, Arts and Sciences, Waseda University, Tokyo, Japan; ^3^Institute of Child Development, University of Minnesota, Minneapolis, MN, United States

**Keywords:** culture, parent socialization, moral development, cross-cultural, parent talk

## Abstract

Moral reasoning develops rapidly in early childhood. Recent evidence from cognitive neuroscience literature suggests that the development of moral reasoning is supported by an integration of cognitive and affective components. However, the role of culture in the development of moral reasoning in young children is under-investigated. Previous cross-cultural research suggests that culture shapes how people interpret other’s behaviors. In particular, people raised in independent cultures, such as the United States, tend to form impressions of others and attribute others’ behaviors to their personal dispositions more quickly than people raised in interdependent cultures, such as Japan. In the present cross-cultural study, we examined parents’ discourse with children in Japan and the United States. Parents and their 3- to 4-year-old children were asked to view and discuss cartoon characters depicting prosocial and antisocial acts. Results indicated that in both cultures, parents discussed about moral actions (e.g., helping, harming) of characters. Furthermore, United States parents were more likely to evaluate dispositional characteristics of characters based on their pro-social and anti-social acts, whereas Japanese parents were more likely to refer to emotion of the characters who got hurt. We discuss implications of cross-cultural differences and similarities in parental moral socialization and the development of moral reasoning in young children.

## Introduction

For over half a century, researchers have suggested that children progress through relative stages in their moral cognition (Nisan and Kohlberg, 1982), from an external control of authority figures to developing an understanding of societal needs and abstract principals of morality (Colby et al., 1983). As children progress through these moral stages, both Piaget and Kohlberg placed a great emphasis on the process in which children update their mental model of morality when they were confronted with moral dilemmas (Walker and Taylor, 1991). Recent studies in moral development with infants and toddlers have shifted from a heavy focus on the confrontation of moral dilemmas and the subsequent reasoning to using visual stimuli based on cartoon or puppet shows that depict pro-social and antisocial actions (Hamlin, 2013). This shift in techniques has allowed for a nonverbal approach to the study of morality, encompassing such groups as preverbal infants, toddlers, preschoolers, and middle childhood individuals.

With this shift in techniques for the study of moral development, researchers have uncovered precursors to more complex moral reasoning that are present potentially as early as three months of age (Hamlin et al., 2011). These methods have also begun to be used in multiple levels of analysis as they eliminate many of the issues that prevented neuroscientific, eye-tracking, psychophysiological, or cross-cultural investigations of developing morality (Cowell and Decety, 2015a; Cowell et al., 2018). Visual characterizations of inter-character harm and help allow for repeated presentations of stimuli, an aspect necessary for neuroscientific studies, and eliminate the necessity of translating and back translating complex moral dilemmas in order to investigate cultural differences.

Utilizing these new character observation based methods, differences have been observed as early as 12 months in the neural processing of helping versus harming toward others (Cowell and Decety, 2015a). These neural computations predict later moral behaviors by toddlerhood (Cowell and Decety, 2015b), and these computations themselves may be related to parental values (Cowell and Decety, 2015a; Meidenbauer et al., 2018; Kim et al., 2021). Using the Chicago Moral Sensitivity task (CMST), one indicated that 12- to 24-month-old children show the difference in the neural processing of prosocial versus antisocial actions as early as 300 ms after stimulus presentation, and the differences present were predicted by parental justice sensitivity (Cowell and Decety, 2015a). Similarly in one study on empathy, preschool children’s empathic processing for those in pain versus those not in pain between 200 and 400 ms post stimulus was predicted by parents empathic values, particularly their empathic concern (Decety et al., 2018). Finally, one recent study indicated that parental justice sensitivity predicted children’s moral neural computations in early components tied to attentional mechanisms (Kim et al., 2021). These burgeoning findings from developmental moral neuroscience suggest a complex interaction between parents’ values, children’s neural computations, children’s behaviors, and the ways in which parents transmit their values to their child. This line of research highlights the necessity of the systematic approach toward understanding the mechanisms through which parents transmit their moral values to their child, including the types of aspects of the scenarios that parents point out to their children.

Historically, little emphasis was placed on the role of parents as socialization agents in the child as scientist model, instead the primary sources of challenge were schools and peers (Walker, 1989). The child as scientist model, as introduced by such researchers as Piaget, argues that the child is active in their own development, seeking out experiences, hypothesizing about the cause and effect relations, and testing these hypotheses, a *de facto* miniature scientist (Piaget, 1955). However, decades of research also highlights the importance of parents and parents socialization techniques in developing children’s social lives including their developing moral sense (Brody and Shaffer, 1982), their interpersonal interactions and their long-term romantic relationship success and social life (Sroufe, 2005), and their internalization of parents’ rules and empathetic concern (Kochanska, 2002). One seminal study suggested that the types of strategies parents use to accommodate or scaffold moral development may be particularly relevant to children’s long-term moral cognition (Walker and Taylor, 1991). In this study, evidence suggested that explicitly measured parental moral reasoning level did not predict children’s moral reasoning level; however, the degree to which parents accommodated the child’s moral reasoning level in their explanations of moral dilemmas predicted long-term moral development level of the child two years later. Recent investigations have explored the process and degree to which parent–child conversations revolves around moral socialization as they are confronted in everyday life (Wainryb and Recchia, 2014). Examining parent–child conversations about real-life moral scenarios, previous research has identified several themes including moral actions or moral behaviors, judgments/evaluations of the overall actions, interpersonal dynamics within the scenario, affective components of the dilemma, and perspective taking/reasoning for the individual (Recchia et al., 2014).

Taken together, this early body of research elucidates the importance of parental discourse and parental attentional queuing strategy in child moral cognitive development. However, the predominant body of research in the field of psychology, and particularly in developmental psychology, has been focused on WEIRD samples, that is, participants who are members of western, educated, industrialized, rich, and democratic societies (Henrich et al., 2010; Nielsen et al., 2017). Accumulating evidence from cultural research demonstrates that cognition is shaped by cultural contexts and shared values, beliefs, and practices (Markus and Kitayama, 1991; Nisbett and Masuda, 2003; Tao et al., 2013). It should be noted that several scholars have argued that there is greater within cultural variability than cross cultural variability (Killen and Wainryb, 2000). Although cultural differences are not necessary represented in individual differences within cultural groups, systematic investigations of cultural differences indicate robust differences at the group level (Na et al., 2010). Particularly, a large body of research has examined cultural differences in attention by comparing East Asian and North American cultural groups using behavioral, eye–tracking, and neurophysiological measurements (Masuda and Nisbett, 2001; Chua et al., 2005; Hedden et al., 2008; Senzaki et al., 2014). Members of North American cultures tend to selectively focus on central objects or people in a scene, whereas members of East Asian cultures holistically attend to both central objects/people and contextual backgrounds (Masuda and Nisbett, 2001; Nisbett and Masuda, 2003). These differences in attention are also involved in social cognition. North Americans tend to attribute others’ behaviors to their internal dispositions and traits compared to East Asians (Choi et al., 1999; Shimizu et al., 2017). For instance, in a study examined neurophysiological underpinnings of spontaneous social evaluation (Na and Kitayama, 2011), European American and Asian American participants were asked to first remember a pair of a facial photo and a trait-implied behavior (e.g., she helped an elderly crossing a street.). When participants were presented with the previously studied photos with the congruent trait words (e.g., kind) and incongruent trait words (e.g., mean) in the following task, only European-Americans exhibited a larger amplitude of N400 for incongruent than congruent traits, suggesting that European Americans are more likely to engage in spontaneous social evaluation than Asian Americans.

Cultural research thus indicates that culture provides an overarching framework that guides individuals’ attention, perception, and evaluation. The degree to which parenting and cultural practices differ is substantial (Bornstein, 2012). Recent studies have demonstrated that parents in the United States and Japan direct their children’s attention differently, as the United States parents direct children’s attention to central objects/characters, whereas Japanese parents direct children’s attention to contextual backgrounds as well as social interactions among characters (Senzaki and Shimizu, 2020, 2022). This type of observation allows for a more naturalistic mapping of parents’ moral discourse with their children and specifically highlights different types of attentional foci associated with parent–child interaction across cultures. In particular, one study (Kuwabara and Smith, 2022) investigated cultural differences in parental discussions of moral action with children. Parents and children in the United States and Japan witnessed one puppet helping or hurting another puppet, and parents were asked to describe the scenarios to their 3- to 4-year-old children. In these interactions, parents in the United States tended to put greater focus on the agent (i.e., helper) or perpetrator (i.e., hinderer) of the moral action with the majority of their discourse aimed at describing the actions, the reasons, or the behaviors of the agent of action. Japanese parents also focused on the helper in prosocial scenes; however, in antisocial scenes, their focus was equally spread toward both the agents and the recipients of action. Such equal allocation of attentional cues from Japanese parents to children may be one index of the cultural emphasis on moral reasoning encompassing social relationships between two characters and the consequence of moral action experienced by both characters. Conversely, United States parents may emphasize the agent of action as internal dispositions and refer to these dispositions in evaluating the scenarios.

The purpose of this study was to extend previous work from developmental and cultural approaches to moral development by investigating parent-child socialization practices in two cultural groups. We used visual cartoon stimuli that have been widely used in examining children’s moral understanding (Cowell and Decety, 2015b). While parents and children observed two characters engaging in either prosocial or antisocial actions, they were asked to discuss their observation. We coded parental speech as an index of their attention foci during socialization of moral development. Verbal descriptions (i.e., speech) have been studied as a behavioral indicator of attention in prior research. For instance, both adults’ and children’s verbal descriptions were correlated to their visual attention measured via an eye-tracker during a scene description task (Senzaki et al., 2014; Senzaki and Shimizu, 2022) as well as during parent–child interaction task (Köster and Kärtner, 2018). Based on previous research investigating parent–child conversations about interpersonal helping and harming in children and adolescents (Recchia et al., 2014), we first analyzed the content of parents’ moral speech focusing on moral action, moral evaluation, and moral emotion. Based on previous cross-cultural research (Kuwabara and Smith, 2022), we also examined the target of attention focusing on the agent (i.e., helper or hinderer) vs. the recipient (i.e., beneficiary of prosocial action or the victim of antisocial action) in parents’ speech. It is important to note that we selected stimuli that depicted clear inter-character harm and help; therefore, we predicted that parents in both cultures were expected to engage their children in moral conversations. Previous research suggests that parents across cultures spontaneously engage their children in moral talk (Wainryb and Recchia, 2014), yet the manifestation of different socialization strategies may differ reflecting unique cultural socialization goals. Therefore, benyond cultural similarities, we also expected cross-cultural differences in parental attention foci as parent-child interactions are embedded in a larger cultural context. First, we expected that parents in the United States would use more moral evaluation than Japanese parents. Second, we predicted that Japanese parents would focus more on the recipient, particularly emotional consequences of the recipient in moral scenarios.

## Materials and Methods

### Participants

Participants were recruited through flyers distributed in childcare and local businesses, and also through advertisements on Social Media and websites as part of a larger study. In this study, 28 children (15 girls, age range = 36–59 months, *M* = 3;6 years, *SD* = 6.18 months; race or ethnicity: 10.7% more than one race, 3.6% Black, 85.7% White) and their parents (26 mothers, 2 fathers) in the United States, and 29 children (17 girls, age range = 36–59 months, *M* = 3;5 years, *SD* = 7.68 months) and their parents (26 mothers, 3 fathers) in Japan participated. The distributions of children’s age and gender were not statistically significant across cultures, *t*(55) = 1.00, *p* > 0.25 and χ^2^(1) = 0.70, *p* > 0.250, respectively. In the United States, all participants were born in the United States and spoke English as their first language. In Japan, all participants identified themselves as Japanese, were born in Japan, and spoke Japanese as their first language. In the United States, 28.6% of parents had an associate degree or some college and 71.4% had a bachelor’s degree. In Japan, 35.5% of parents had an associate degree or some college and 64.5% had a bachelor’s degree. Parents’ age and race or ethnicity variables were not recorded.

### Study Description

The stimuli were adapted from the Chicago moral sensitivity task (Cowell and Decety, 2015b). Parent–child dyads were shown three picture sequence of characters (shapes with arms and legs) performing prosocial or antisocial actions. In three images, one character (hereafter referred to as agent) engaged in a prosocial (e.g., helping) or antisocial (e.g., pushing) action toward the other character (hereafter referred to as recipient). Each picture was presented for 1 s at a time on the screen to allow parents to see the entire sequence, then all three pictures appeared on the screen for a total of 30 s for the parent to describe the scenarios and talk with their child about their observation. Parents were instructed to talk with their children as naturally as possible. Prosocial and antisocial scenarios were matched, and they were presented in a random order. In the United States, participants observed two sets of prosocial and antisocial scenarios and in Japan, participants observed three sets of prosocial and antisocial scenarios. The number of scenarios observed in two countries was different because the United States data collection occurred first. After the Japanese data collection, the intention was to test a comparable sample in the United States, but COVID testing restrictions prevented such matched procedural testing. All dependent variables regarding parental speech are thus represented as proportions of overall utterances for standardization within country and due to this inherent testing difference.

### Coding

The coding unit consisted of a proposition, which was defined as a subject–verb construction with a unique or implied verb (Fivush et al., 1995). For example, “He was nice” was one proposition and “He was nice and helpful” was two. In this study, we focused on parental socialization techniques; thus, we coded parents’ contributions in moral talk. Prosocial and antisocial scenarios were coded separately. From the previous research (Recchia et al., 2014; Shimizu et al., 2018), a coding scheme has been developed to analyze parent–child conversations. First, parents’ narratives were coded into three moral attentional foci: moral action, moral evaluation, and moral emotion (Table 1), and non-moral speech. Parents’ narratives were further coded whether the description was directed toward the agent, recipient, or others. These coding categories were mutually exclusive, and the frequency was counted for each dyad. Because the number of scenarios differed between two cultural groups, we report the proportion data using the total number of utterances. Coding was completed in the original language. In each culture, two coders who were unaware of the study hypotheses were trained in repeated joint coding sessions using a subset of 10% of the narratives. Two coders in each country independently coded an overlapping 20% of the data to calculate the intercoder reliability. Intercoder agreement (Cohen’s kappa) was 0.84 for the United States data and 0.87 for the Japanese data. Finally, 20% of the Japanese transcripts were randomly selected and translated into English, which was coded by an English-speaking coder. The intercoder reliability reached 0.85 for this subset of Japanese transcripts translated and coded in English.

**TABLE 1:** Parental moral speech focus coding category, definition, and example.

Coding category	Definition	Example
Moral action	Parents describe moral actions or behaviors	“He was pushing.”; “The triangle was pushed over.”; “The square was helping.”; “She was given a cupcake.”
Moral evaluation	Parents provide positive or negative evaluation	“She is nice.”; “That was kind of the red one.”; “Square is mean.”; “That wasn’t very nice.”
Moral emotion	Parents use emotional or psychological states in their description	“The triangle is sad.”; “I feel sorry for him.”; “She looks happy.”; “What is she feeling?”

## Results

### Cross-Cultural Differences and Similarities in Parental Attentional Focus

To investigate the cultural differences in parental attention focus, a 2 (Culture: United States/Japan) × 2 (Moral scenario: Antisocial/Prosocial) × 3 (Focus: action, emotion, evaluation) Factorial Analysis of variance with proportion of talk as the dependent variable was used, with Bonferonni corrections applied to all post-hoc comparisons to account for family-wise error.

A main effect of focus was observed [*F* (2, 55) = 37.36, *p* < 0.001, η_p_^2^ = 0.405], where the most commonly used parental moral speech focus was toward the moral action itself (*M* = 0.272, 95% CI: 0.244, 0.330). This strategy was used significantly more often than appeals toward the emotions of the characters (*M* = 0.145, CI: 0.117, 0.172 or toward evaluations of characters (*M* = 0.115, CI: 0.244, 0.330) and the usage of these two strategies was not significantly different. There were no significant main effects of culture [*F* (1, 56) = 0.06, *n.s.*], moral scenario [*F* (1, 56) = 0.007, *n.s.*], nor a significant three-way interaction.

**FIGURE 1:**
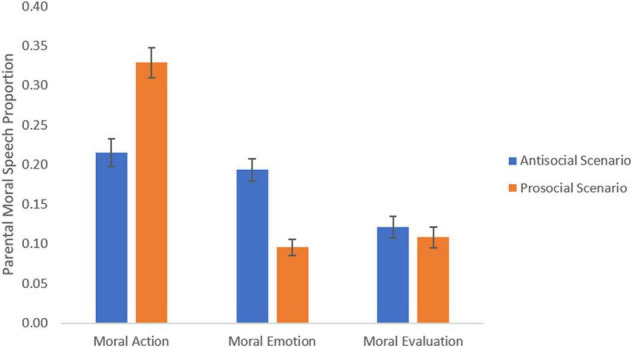
Moral attentional foci in parental narratives as a function of moral scenario. Error bars represent the ± 1 standard error (SE).

There was a significant moral scenario x focus interaction [*F* (1, 56) = 34.15, *p* < 0.001, η_p_^2^ = 0.38]. In decomposing this interaction, parental attentional cues in antisocial scenarios were marked by greater use of moral action (*M* = 0.215, CI: 0.179, 0.251) than evaluation (*M* = 0.121, CI: 0.094, 0.148, Bonferroni-corrected *p* < 0.01) and more emotion cues (*M* = 0.194, CI: 0.165, 0.222 than evaluation cues (Bonferroni-corrected *p* < 0.001). Parental attentional cues in prosocial scenarios were marked by greater use of moral action (*M* = 0.329, CI: 0.291, 0.366) than emotion (*M* = 0.095, CI: 0.075, 0.116) and evaluation (*M* = 0.108, CI: 0.082, 0.134, Bonferroni-corrected *p* < 0.001 and *p* < 0.001, respectively), but no differences were observed in the use of emotion versus evaluation (Figure 1).

**FIGURE 2:**
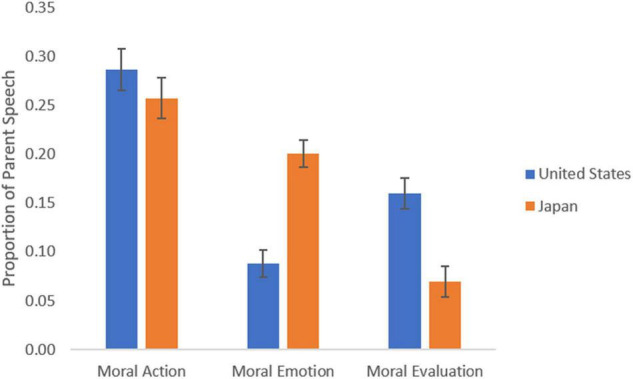
Cross-cultural differences in moral attentional foci in parental narratives. Error bars represent the ± 1 standard error (SE).

Cultural differences in parental focus were observed (as evidenced by a significant culture × focus interaction [*F* (1, 56) = 14.60, *p* < 0.001, η_p_^2^ = 0.210], wherein parents in Japan and the United States both used action focuses relatively equally (*M* = 0.257, CI: 0.215, 0.300; *M* = 0.287, CI: 0.244, 0.330, respectively), but Japanese parents were more likely to use speech that focused on emotions (*p* < 0.001) than United States parents (*M* = 0.201, CI: 0.173, 0.229; *M* = 0.088, CI: 0.060, 0.116, respectively), whereas United States parents were more likely to use speech focusing on an evaluation/judgment (*p* < 0.001) than Japanese parents (*M* = 0.160, CI: 0.128, 0.192; *M* = 0.069, CI: 0.038, 0.101, respectively, Figure 2).

### Parental Recipient Versus Agent Morally Directed Speech

To investigate and replicate previous findings regarding if there were cultural and moral differences in the degree to which parents referenced recipients versus agents in their speech, a 2 (United States/Japan) × 2 (Antisocial/Prosocial) × 2 (Recipient/Agent) factorial ANOVA with proportion of parental speech as the dependent variable was conducted, with *post hoc* decompositions of significant effects using a Bonferroni correction for multiple comparisons.

**FIGURE 3:**
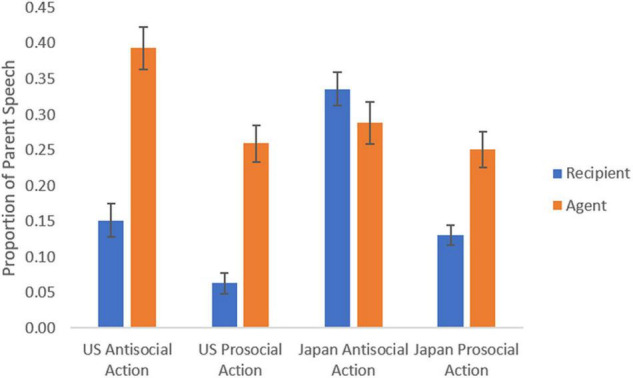
Cross-cultural differences in attention of target character as a function of moral scenario. Error bars represent the ± 1 standard error (SE).

A significant main effect of character type (recipient/agent) was observed [*F* (1, 56) = 40.14, *p* < 0.001, η_p_^2^ = 0.422], with more parental speech directed toward the agent than the recipient (agent *M* = 0.297, CI: 0.267, 0.328; recipient *M* = 0.174, CI: 0.153, 0.194). There was also a significant interaction effect of culture x character [*F* (1, 56) = 23.53, *p* < 0.001, η_p_^2^ = 0.30], wherein United States parents spoke significantly more about the agent than the recipient (*p* < 0.001, agent *M* = 0.326, CI: 0.283, 0.370; recipient *M* = 0.108, CI: 0.078, 0.137), but Japanese parents did not significantly differ in the speech directed toward recipient versus agents (*n.s.*, agent *M* = 0.269, CI: 0.226, 0.311; recipient *M* = 0.240, CI: 0.210, 0.269). Importantly for interpretation, these effects were characterized by a significant three-way interaction [*F* (1, 56) = 10.66, *p* < 0.01, η_p_^2^ = 0.162], wherein United States parents were more likely to talk about the agent than the recipient regardless of moral scenarios (agent *M* = 0.326, CI: 0.283, 0.370; recipient *M* = 0.108, CI: 0.078, 0.137), but Japanese parents only showed this effect in the prosocial scene (agent *M* = 0.251, CI: 0.200, 0.301; recipient *M* = 0.139, CI: 0.109, 0.169), and Japanese parents talked about both the agent and recipient equally in antisocial scenarios (*n.s.*, agent *M* = 0.286, CI: 0.227, 0.345; recipient *M* = 0.340, CI: 0.294, 0.387) (Figure 3).

## Discussion

These results demonstrate both cultural similarities and differences in caregivers attentional focus and cueing regarding moral socialization. First, in both cultures, caregivers were most likely to use moral action descriptions while cueing joint attention to the scenarios, such as “The square is helping the triangle.” or “The oval is pushing the square.” Given that moral development is typically a central part of culture (Shweder et al., 1987) it is not surprising that this pattern was similar across cultures. We predicted that caregivers would discuss moral actions as primary attentional cues because we used scenarios that depicted clear and prototypical antisocial vs. prosocial behaviors, such as interpersonal harm. Furthermore, in our previous research, we found similar preferential reaching behaviors in United States and Japanese infants (Shimizu et al., 2018) and similar attentional sensitivity measured via eye-tracking among United States and Japanese preschoolers (Shimizu et al., 2021). Together, these results suggest that teaching morality through attentional cueing is a common practice engaged by caregivers of young children in both cultures, and young children demonstrate sensitivity to others’ moral actions.

In addition to cultural similarities, we also found significant cross-cultural differences in caregiver moral descriptors beyond moral action. As predicted, the United States, caregivers were more likely to use evaluative terms (e.g., “Square was nice” or “Oval was mean.”) than Japanese caregivers. This finding replicated our previous study that examined socialization of mothers to preverbal infants in the United States and Japan (Shimizu et al., 2018). Previous cross-cultural research has demonstrated systematic and substantial differences in attentional patterns between members of East Asian and North American cultures (Markus and Kitayama, 1991; Nisbett and Masuda, 2003). Recent studies have further examined socialization practices that foster children to develop culturally unique patterns of attention (Senzaki and Shimizu, 2020, 2022; Kuwabara and Smith, 2022). The present research adds to this body of work by demonstrating culturally divergent parental discourses and associated attentional foci in socio-cognitive and moral aspects of development.

The results of the present study also demonstrated that Japanese caregivers were more likely to use emotion references (e.g., “Triangle looks very sad.” “Now square is happy.”) than did United States caregivers. These results provide support for previous theories indicating cultural uniqueness in moral reasoning. While the most influential theory of moral development proposed by Kohlberg focuses on justice orientation of morality, morality of Japanese people has been characterized as moral particularism, which prioritizes interpersonal relationships and situation-specific moral values (Benedict, 1948; Naito, 1994). For example, Reischauer writes “the Japanese on the whole do think less in terms of abstract ethical principles than do Westerners and more in terms of concrete situations and human feelings” (Reischauer, 1977, p. 140). More recent cross-cultural studies interviewed parents and preschool teachers suggest that independence is emphasized as one of the most important skills for children to develop in the United States (Azuma and Kashiwagi, 1987; Wu and Davidson, 1989; Hayashi et al., 2009; Yasumoto, 2010). On the other hand, Japanese parents and preschool teachers reported that empathy (or *omoiyari* in Japanese) is critical for Japanese children’s character development. *Omoiyari* is defined as “the ability and willingness to feel what others are feeling, to vicariously experience the pleasure and pain that they are undergoing, and to help them satisfy their wishes” (Lebra, 1976). These cultural differences are reflected in the findings of the present study, as Japanese caregivers emphasized the emotion and feelings of the recipient, especially in antisocial scenarios where the recipient was hurt.

Lastly, we found that United States and Japanese caregivers attended to the characters differently. In the United States, parents used more moral speech in the antisocial scenes than prosocial scenes. This finding is similar to the ways parents talk about children’s own moral behaviors (Recchia et al., 2014). Furthermore, the United States caregivers directed their moral speech to agents in the event who was acting prosocially or antisocially compared to the recipient of the action, regardless of moral scenarios. This finding replicated a recent previous research with United States and Japanese caregivers (Kuwabara and Smith, 2022). In Japan, caregivers focus to agent and recipient changed depending on moral scenarios. In the prosocial scene, Japanese caregivers used more moral speech toward agents over recipients, whereas caregivers’ moral speech was distributed equally to agents and recipients in antisocial scenarios. This cultural difference can be attributed to the higher rate of emotion words used by Japanese caregivers.

The present findings provide evidence for the role of culture demonstrating that parent-child interaction is embedded in the larger cultural context and prioritized moral considerations prevalent in different cultural groups (Rogoff, 2003; Miller, 2005). Parental attentional cueing strategies are likely foundational support for moral development in children. In both cultures, parents used moral action speech most commonly, and this may relate to an early development of identification of interpersonal helping and harming, as indicated in a previous research demonstrating that children exhibit such a differentiation in neural activities as early as 300 ms (Decety and Cowell, 2018). Alternatively, some have argued that continuing practice both by the child and scaffolding by parents of moral evaluation would yield more complex understandings of the reasons for good and bad actions, leading to a more robust moral reasoning (Turiel, 1966). While this approach has been widely applied in developmental research, the findings of the present research and other cross-cultural studies (Shimizu et al., 2018) question the possibility of overgeneralization of culturally specific aspects of moral development. cross-cultural studies have well documented the tendency of people in Western cultures to emphasize dispositional attributions as a cause of one’s action, while East Asians tend to incorporate both internal dispositions and situational factors (Choi et al., 1999). Such cultural differences in social cognition explain how Japanese parents in the present study avoided explicit evaluation and instead focused their attention to the consequences of moral action and further discussed emotional reactions of the recipient of antisocial actions. Future research should investigate the relation between parent-child moral socialization and the long-term development of morality.

While our results shed light on the complex and nuanced role of culture in socialization of morality by identifying both cultural similarities and differences, there are several limitations of the current study. First, the current study did not examine the relation between socialization and children’s moral development. Recent studies have demonstrated that caregivers’ sensitivity to justice orientation predicts children’s neural differentiation of prosocial vs. antisocial behaviors (Cowell and Decety, 2015a; Kim et al., 2021). Future study should investigate the relation between neural development in children and caregiver-child socialization. Another limitation is that we only included clear moral scenarios that involved interpersonal harm between two characters. Children’s understanding of morality varies across cultures when moral or transgressional acts benefit or harm individuals vs. groups (Fu et al., 2016). Moreover, some evidence suggests that children and adolescents’ neural processing and prosocial behaviors were predicted by parents values about equity and equality using nuanced moral scenarios including the need of the recipients and resource importance (Meidenbauer et al., 2018). In order to further understand the role of culture and socialization in the development morality, a diverse range of scenarios should be used in future research. Finally, it is important to note that our sample consisted of highly educated parents in the United States and Japan. As previous research suggests the influence of socio-economic and other cultural factors on moral reasoning (e.g., Haidt et al., 1993; Caravita et al., 2012), future research should examine moral socialization with more diverse populations with bigger samples.

In summary, our findings provide evidence for culturally similar and specific attentional focus and parental moral socialization strategies in two cultures. In the process of teaching, narratives are essential in creating meaning (Bruner, 1990). Caregivers help children understand their social world by providing culturally shared behavioral scripts; thus, caregivers play a key role in carrying forward a cultural tradition by sharing their understandings and practices with their children (Shweder et al., 2006). The present findings shed light on the potential role of parental socialization in the development of morality across cultures.

## Data Availability Statement

The raw data supporting the conclusions of this article will be made available by the authors, without undue reservation.

## Ethics Statement

The studies involving human participants were reviewed and approved by Institutional Review Board, University of Wisconsin–Green Bay. Written informed consent to participate in this study was provided by the participants’ legal guardian/next of kin.

## Author Contributions

SS and JC conceived and designed the experiments, analyzed the data, and wrote the manuscript. SS, JC, YS, and DC-B performed the experiments. All authors reviewed the manuscript for submission.

## Conflict of Interest

The authors declare that the research was conducted in the absence of any commercial or financial relationships that could be construed as a potential conflict of interest.

## Publisher’s Note

All claims expressed in this article are solely those of the authors and do not necessarily represent those of their affiliated organizations, or those of the publisher, the editors and the reviewers. Any product that may be evaluated in this article, or claim that may be made by its manufacturer, is not guaranteed or endorsed by the publisher.

## References

[B1] Azuma H. Kashiwagi K. (1987). Descriptors for an intelligent person: a Japanese study. *Jpn. Psychol. Res.* 29 17–26. 10.4992/psycholres1954.29.17

[B2] Benedict R. (1948). Anthropology and the humanities. *Am. Anthropol.* 50 585–593. 18128881 10.1525/aa.1948.50.4.02a00020

[B3] Bornstein M. H. (2012). Cultural approaches to parenting. *Parent Sci. Pract.* 12 212–221. 10.1080/15295192.2012.683359.Cultural 22962544 PMC3433059

[B4] Brody G. H. Shaffer D. R. (1982). Contributions of parents and peers to children’s moral socialization. *Dev. Rev.* 2 31–75.

[B5] Bruner J. (1990). *Acts of Meaning.* Cambridge, MA: Harvard University Press.

[B6] Caravita SC. Giardino S. Lenzi L. Salvaterra M. Antonietti A. (2012). Socio-economic factors related to moral reasoning in childhood and adolescence: the missing link between brain and behavior. *Front.Hum. Neurosci.* 6:262. 10.3389/fnhum.2012.00262 23015787 PMC3449439

[B7] Choi I. Nisbett R.E. Norenzayan A. (1999). Causal attribution across cultures: Variation and Universality. *Psychol. Bull*. 125, 47–63

[B8] Chua H. F. Boland J. E. Nisbett R. E. (2005). Cultural variation in eye movements during scene perception. *Proc. Natl. Acad. Sci. U.S.A* 102 12629–12633. 10.1073/pnas.0506162102 16116075 PMC1194960

[B9] Colby A. Kohlberg L. Gibbs J. Lieberman M. Fischer K. Saltzstein H. D. (1983). A longitudinal study of moral judgment. *Monogr. Soc. Res. Child Dev.* 48:124.

[B10] Cowell J. M. Decety J. (2015a). Precursors to morality in development as a complex interplay between neural, socioenvironmental, and behavioral facets. *Proc. Natl. Acad. Sci. U.S.A* 112 12657–12662. 10.1073/pnas.1508832112 26324885 PMC4611595

[B11] Cowell J. M. Decety J. (2015b). The neuroscience of implicit moral evaluation and its relation to generosity in early childhood. *Curr. Biol.* 25 93–97. 10.1016/j.cub.2014.11.002 25532892 PMC4286511

[B12] Cowell J. M. Calma-Birling D. Decety J. (2018). Domain-general neural computations underlying prosociality during infancy and early childhood. *Curr. Opin. Psychol.* 20 66–71. 10.1016/j.copsyc.2017.08.016 28863354

[B13] Decety J. Cowell J. M. (2018). Interpersonal harm aversion as a necessary foundation for morality: a developmental neuroscience perspective. *Dev. Psychopathol.* 30 153–164. 10.1017/S0954579417000530 28420449

[B14] Decety J. Meidenbauer K. L. Cowell J. M. (2018). The development of cognitive empathy and concern in preschool children: a behavioral neuroscience investigation. *Dev. Sci.* 21:e12570. 10.1111/desc.12570 28523733

[B15] Fivush R. Haden C. Adam S. (1995). Structure and coherence of preschoolers’ personal narratives over time: implications for childhood amnesia. *J. Exp. Child Psychol.* 60 32–56.

[B16] Fu G. Heyman G. D. Lee K. (2016). Learning to be unsung heroes: development of reputation management in two cultures. *Child Dev.* 87 689–699. 10.1111/cdev.12494 27189397

[B17] Haidt J. Koller S. H. Dias M. G. (1993). Affect, culture, and morality, or is it wrong to eat your dog? *J. Pers. Soc. Psychol* 65 613–628. 10.1037/0022-3514.65.4.613 8229648

[B18] Hamlin J. K. (2013). Moral judgment and action in preverbal infants and toddlers: evidence for an innate moral core. *Curr. Dir. Psychol. Sci.* 22 186–193. 10.1177/0963721412470687

[B19] Hamlin J. K. Wynn K. Bloom P. (2011). 3-month-olds show a negativity bias in their social evaluations. *Dev. Sci.* 13 923–929. 10.1111/j.1467-7687.2010.00951.x.3-month-olds PMC2966030 20977563

[B20] Hayashi A. Karasawa M. Tobin J. (2009). The Japanese preschool ‘ s pedagogy of feeling: cultural strategies for supporting young children’s emotional development preface: sad fish and lonely carrots. *Ethos* 37 32–49. 10.1111/j.1548-1352.2009.01030.x.JAPANESE

[B21] Hedden T. Ketay S. Aron A. Markus H. R. Gabrieli J. D. E. (2008). Cultural influences on neural substrates of attentional control. *Psychol. Sci.* 19 12–17. 18181784 10.1111/j.1467-9280.2008.02038.x

[B22] Henrich J. Heine S. J. Norenzayan A. (2010). The weirdest people in the world? *Behav. Brain Sci.* 33 61–83. 20550733 10.1017/S0140525X0999152X

[B23] Killen M. Wainryb C. (2000). Independence and interdependence in diverse cultural contexts. *New Dir. Child Adolesc. Dev.* 87, 5–21. 10.1002/cd.23220008703 10763564

[B24] Kim M. Decety J. Wu L. Baek S. Sankey D. (2021). Neural computations in children’s third-party interventions are modulated by their parents’ moral values. *NPJ Sci. Learn.* 6 1–13. 10.1038/s41539-021-00116-5 34921148 PMC8683432

[B25] Kochanska G. (2002). Between mothers and their young children: a context for the early development of conscience. *Curr. Dir. Psychol. Sci.* 11 191–195. 10.1111/1467-8721.00198

[B26] Köster M. Kärtner J. (2018). Context-sensitive attention is socialized via a verbal route in the parent-child interaction. *PLoS One* 13:e0207113. 10.1371/journal.pone.0207113 30408099 PMC6224102

[B27] Kuwabara M. Smith L. B. (2022). Focus on one or more? Cultural similarities and differences in how parents talk about social events to preschool children. *Front. Psychol.* 12:778960. 10.3389/fpsyg.2021.778960 35058848 PMC8764154

[B28] Lebra T. (1976). *Japanese Patterns of Behavior.* Honolulu: University of Hawaii Press.

[B29] Markus H. R. Kitayama S. (1991). Culture and the self: implications for cognition, emotion, and motivation. *Psychol. Rev.* 98 224–253. 10.1037//0033-295X.98.2.224

[B30] Masuda T. Nisbett R. E. (2001). Attending holistically versus analytically: comparing the context sensitivity of Japanese and Americans. *J. Pers. Soc. Psychol.* 81:922. 10.1037//0022-3514.81.5.922 11708567

[B31] Meidenbauer K. L. Cowell J. M. Killen M. Decety J. (2018). A developmental neuroscience study of moral decision-making regarding resource allocation. *Child Dev.* 89 1177–1192. 10.1111/cdev.12698 27982418

[B32] Miller J. G. (2005). Essential role of culture in developmental psychology. *New Dir. Child Adolesc. Dev.* 2005 33–41. 10.1002/cd.135 16342891

[B33] Na J. Kitayama S. (2011). Spontaneous trait inference is culture-specific: behavioral and neural evidence. *Psychol. Sci.* 22 1025–1032. 10.1177/0956797611414727 21737573

[B34] Na J. Grossmann I. Varnum M. E. W. Kitayama S. Gonzalez R. Nisbett R. E. (2010). Cultural differences are not always reducible to individual differences. *Proc. Natl. Acad. Sci. U.S.A* 107 6192–6197. 10.1073/PNAS.1001911107 20308553 PMC2851996

[B35] Naito T. (1994). A survey of research on moral development in Japan. *Cross cult. Res.* 28 40–57.

[B36] Nielsen M. Haun D. Kärtner J. Legare C. H. (2017). The persistent sampling bias in developmental psychology: a call to action. *J. Exp. Child Psychol.* 162 31–38. 10.1016/j.jecp.2017.04.017 28575664 PMC10675994

[B37] Nisan M. Kohlberg L. (1982). Universality and variation in moral judgment: a longitudinal and cross-sectional study in Turkey. *Child Dev.* 53 865–876.

[B38] Nisbett R. E. Masuda T. (2003). Culture and point of view. *Proc. Natl. Acad. Sci. U.S.A* 100 11163–11170. 12960375 10.1073/pnas.1934527100 PMC196945

[B39] Piaget J. (1955). *The child’s construction of reality.* Abingdon: Routledge & Paul.

[B40] Recchia H. E. Wainryb C. Bourne S. Pasupathi M. (2014). The construction of moral agency in mother-child conversations about helping and hurting across childhood and adolescence. *Dev. Psychol.* 50 34–44. 10.1037/a0033492 23795554

[B41] Reischauer E. O. (1977). *The Japanese, Charles E.* Tokyo: Tuttle Co.

[B42] Rogoff B. (2003). *The Cultural Nature of Human Development.* Oxford: Oxford university press.

[B43] Senzaki S. Shimizu Y. (2020). Early learning environments for the development of attention: Maternal narratives in the United States and Japan. *J. Cross. Cult. Psychol.* 51, 187–202. 10.1177/0022022120910804 33311733 PMC7727458

[B44] Senzaki S. Shimizu Y. (2022). Different types of focus: caregiver-child interaction and changes in preschool children’s attention in two cultures. *Child Dev.* epub ahead of press, 10.1111/cdev.13731 35098526

[B45] Senzaki S. Masuda T. Nand K. (2014). Holistic versus analytic expressions in artworks: cross-cultural differences and similarities in drawings and collages by Canadian and Japanese school-age children. *J. Cross Cult. Psychol.* 45 1297–1316.

[B46] Shimizu Y. Lee H. Uleman J. S. (2017). Culture as automatic processes for making meaning: spontaneous trait inferences. *J. Exp. Soc. Psychol.* 69 79–85. 10.1016/j.jesp.2016.08.003

[B47] Shimizu Y. Senzaki S. Cowell J. M. (2021). Cultural similarities and differences in the development of sociomoral judgments: an eye-tracking study. *Cogn. Dev.* 57:100974. 10.1016/J.COGDEV.2020.100974 33380770 PMC7769212

[B48] Shimizu Y. Senzaki S. Uleman J. S. (2018). The influence of maternal socialization on infants’ social evaluation in two cultures. *Infancy* 23 748–766. 10.1111/infa.12240

[B49] Shweder R. A. Goodnow J. J. Hatano G. LeVine R. A. Markus H. R. Miller P. J. (2006). The cultural psychology of development: one mind, many mentalities. In Damon W. Lerner R. M. (Eds.), *Handbook of child psychology* (716-792). New Jersey, NJ: John Wiley & Sons.

[B50] Shweder R. A. Mahapatra M. Miller J. G. (1987). “Culture and moral development,” in *The Emergence of Morality in Young Children*, eds Kagan J. Lamb S. (Chicago: University of Chicago Press). 1–75.

[B51] Sroufe L. A. (2005). Attachment and development: a prospective, longitudinal study from birth to adulthood. *Attach. Hum. Dev.* 7 349–367. 10.1080/14616730500365928 16332580

[B52] Tao A. Zhou Q. Lau N. Liu H. (2013). Chinese American immigrant mothers’ discussion of emotion with children: relations to cultural orientations. *J. Cross Cult. Psychol.* 44 478–501.

[B53] Turiel E. (1966). An experimental test of the sequentiality of developmental stages in the child’s moral judgments. *J. Pers. Soc. Psychol.* 3 611–618. 10.1037/h0023280 5938996

[B54] Wainryb C. Recchia H. E. (2014). *Talking About Right and Wrong: Parent-child Conversations as Contexts for Moral Development.* Cambridge, MA: Cambridge University Press.

[B55] Walker L. J. (1989). A longitudinal study of moral reasoning. *Child Dev.* 60 157–166. 2702866 10.1111/j.1467-8624.1989.tb02705.x

[B56] Walker L. J. Taylor J. H. (1991). Family interactions and the development of moral reasoning. *Child Dev.* 62 264–283.

[B57] Wu T. J. D. Davidson D. (1989). *Preschool in Three Cultures.* New Haven: Yale University Press.

[B58] Yasumoto S. (2010). *Culture, Cognition, and Parenthood in Japanese and American Homes. Dissertation, Georgia State University.* Atlanta, GA: Georgia State University.

